# TRIB1 rs17321515 and rs2954029 gene polymorphisms increase the risk of non-alcoholic fatty liver disease in Chinese Han population

**DOI:** 10.1186/s12944-019-1001-z

**Published:** 2019-03-09

**Authors:** Qun Liu, Feng Xue, Jing Meng, Shou-Sheng Liu, Li-Zhen Chen, Hui Gao, Ning Geng, Wen-Wen Jin, Yong-Ning Xin, Shi-Ying Xuan

**Affiliations:** 1 Medical College of Qingdao University, Qingdao, 266071 China; 2 Department of Infectious Disease, Qingdao Municipal Hospital, Qingdao, 266011 China; 3 Department of Gastroenterology, Qingdao Municipal Hospital, 1 Jiaozhou Road, Qingdao, 266011 Shandong Province China; 4 Central Laboratories, Qingdao Municipal Hospital, Qingdao, 266071 China; 5 Digestive Disease Key Laboratory of Qingdao, Qingdao, 266071 China

**Keywords:** Non-alcoholic fatty liver disease, TRIB1, Polymorphism, lipids metabolism

## Abstract

**Background:**

Dysregulation of the lipid homeostasis is an independent risk factor for non-alcoholic fatty liver disease (NAFLD). Some studies had demonstrated that *TRIB1* gene polymorphisms affect the plasma lipids metabolism, but no related data was available for *TRIB1* gene polymorphisms in the lipids metabolism in Chinses Han population. The present study was conducted to investigate the association between *TRIB1* gene polymorphisms (rs17321515 and rs2954029) and the risk of NAFLD in Chinese Han population and their effects on serum lipid profiles.

**Patients and methods:**

*TRIB1* rs17321515 and rs2954029 gene polymorphisms were genotyped using the polymerase chain reaction (PCR) in B-type ultrasonography-proven NAFLD patients (*n* = 146) and healthy controls (*n* = 175). Serum lipid profiles were determined using biochemical methods. Statistical analyses were performed using SPSS 22.0 statistical software.

**Results:**

The allele distributions of *TRIB1* rs17321515 A and rs2954029 A were significant different between the NAFLD patients and healthy controls (*P* = 0.026, *P* = 0.045, respectively). The genotype distribution of *TRIB1* rs17321515 was significant different between NAFLD patients and healthy controls (*P* = 0.038). The TRIB1 rs17321515 GA + AA genotype and TRIB1 rs2954029 TA + AA genotype markedly increase the NAFLD risk (OR = 1.885; 95%CI: 1.157–3.070; OR = 1.627; 95%CI: 1.011–2.619, respectively), after adjusted for age, gender, and body mass index, the NAFLD risk still significant (OR = 2.240; 95%CI: 1.196–4.197; OR = 2.050; 95%CI: 1.110–3.786, respectively). In addition, *TRIB1* rs17321515 A and rs2954029 A carriers possess the higher lipid profiles in the included subjects.

**Conclusions:**

*TRIB1* rs17321515 and rs2954029 were significant associated with the risk of NAFLD in Chinese Han population. The rs17321515 A and rs2954029 A allele increases the serum lipid profiles in Chinese Han population.

## Background

Non-alcoholic fatty liver disease (NAFLD), the most common form of chronic liver diseases, is now recognized as a severe public health concern in the worldwide [1]. The overall prevalence of NAFLD is approximately 25 and 27% in the world and Asian, respectively [2, 3]. Established risk factors for NAFLD including aging, insulin resistance, hyperlipidemia, obesity, type 2 diabetes, dysregulation of the lipid homeostasis, and genetic factors, and the prevalence of NAFLD is increased with the increasing body mass index [4–8]. Biopsy is still regarded as the diagnostic gold standard for NAFLD, but the deficiency of biopsy such as invasiveness and possible sample error remain could be ignored [9, 10]. In recent years, some non-invasive, genetics-based clinical diagnostic methods for NAFLD had been developed. Leon-Mimila et al. reported a genetic risk score system that could evaluate the risk of NASH by combining the variant alleles of *PNPLA3* rs738409, *LYPLAL1* rs12137855, *GCKR* rs1260326, and *PPP1R3B* rs4240624 [11]. Accumulated studies emphasized that finding the new SNPs site which associated with NAFLD is very meaningful for the prediction and diagnosis of NAFLD.

Tribbles-1 (TRIB1) is one of the members of tribbles family that was first identified in *Drosophila* and possesses the function to regulate the cell division and migration [12, 13]. In 2008, genome-wide association studies (GWAS) first revealed the association of *TRIB1* rs17321515 (A > G) and rs2954029 (A > T) gene polymorphisms with the serum level of triglycerides (TG) [14, 15]. Subsequent abundant studies had shown that *TRIB1* rs17321515 and rs2954029 were significant associated with the dysregulation of serum lipids levels and the risk of cardiovascular disease. Tai et al. investigated the effect of *TRIB1* rs17321515 in Malay population, the results showed that *TRIB1* rs17321515 was significant associated with the elevated level of total cholesterol (TC) and low density lipoprotein (LDL), and the risk of cardiovascular disease (CVD) [16]. The elevated serum TG level and risk of coronary heart disease in *TRIB1* rs17321515 A allele carriers in Chinese Han population were also be observed [17]. Varbo et al. conducted a study to test the effect of *TRIB1* rs2954029 in the general population, the results showed that *TRIB1* rs2954029 A allele carriers had the higher serum levels of TG, TC, apolipoprotein B, and LDL-C, and the lower high density lipoprotein (HDL) than A allele non-carriers [18]. The similar effect of *TRIB1* rs2954029 in lipids metabolism was also observed in Japanese community-dwelling women, which showed an elevated TG concentration in AA genotype carriers [19].

There is no doubt about the significant role of *TRIB1* rs17321515 and rs2954029 in the lipid metabolism and the risk of cardiovascular disease. In consideration of the tight association of NAFLD with the dysregulation of lipids metabolism and the risk of cardiovascular disease [20–22], it is meaningful to explore the effect of *TRIB1* rs17321515 and rs2954029 in the NAFLD patients. The purpose of this study was to investigate the association of *TRIB1* rs17321515 and rs2954029 with the risk of NAFLD and their effects on the lipid metabolism in Chinese Han population.

## Patients and methods

### Study subjects

This case-control study was approved by the Ethical Committee of Qingdao Municipal Hospital (Qingdao, China). This study was carried out in accordance with the principles of the declaration of Helsinki and its appendices [23]. All the subjects had written the informed consent before participated in this study.

From June 2018 to November 2018, 146 NAFLD patients diagnosed by B-type ultrasonography (65 females and 81 males, mean age 61.08 ± 15.70 years) and 175 B-type ultrasonography confirmed healthy controls that matched for sex and age (87 females and 88 males, mean age 60.15 ± 11.96 years) were enrolled in this study. The NAFLD patients were collected from the Department of Gastroenterology and the healthy controls were collected from the Medical Center of Qingdao Municipal Hospital. All the individuals were unrelated Northern Han Chinese origin. The diagnosis of NAFLD was performed according to the guidelines of American association for the study of liver diseases (AASLD) [24]. Subjects with other causes of liver disease were excluded, including excess alcohol intake (≥ 210 g/week for males and ≥ 140 g/week for females), chronic viral hepatitis, drug-induced liver injury, Wilson’s disease, alphal-antitrypsin deficiency, viral and autoimmune hepatitis, alphal-antitrypsin deficiency and alpha-l-antitrypsin deficiency. The healthy controls were confirmed by the same examination at Qingdao Municipal hospital.

### Biochemical analyses

The basic clinical information (name, gender, age, body height, and weight) were obtained by a standard study questionnaire. The body mass index (BMI) of each subject was calculated equals to mass (kg)/height (m)^2^. Blood sample was collected from median vein of each subject after a 12-h overnight, and the blood sample was placed into an ethylene diamine tetraacetic acid (EDTA)-containing tube. The serum levels of alanine aminotransferase (ALT), aspartate aminotransferase (AST), gamma-glutamyltranspeptidase (GGT), alkaline phosphatase (ALP), triglyceride (TG), total cholesterol (TC), high-density lipoprotein (HDL), low-density lipoprotein (LDL), total bilirubin (TBIL), fasting plasma glucose (FPG) were measured by standard clinical laboratory techniques (IChem-540 automatic biochemical analyzer, Shenzhen, China), respectively. Environmental factors were excluded in the study.

### Genomic DNA extraction and genotyping

Blood genomic DNA was isolated with a genomic DNA purification kit (TIANGEN, Beijing, China) according to the manufacturer’s instructions and stored at − 20 °C until use. The genotyping of 2 SNPs, rs17321515 and rs2954029, in the TRIB1 gene was conducted using the polymerase chain reaction (PCR). Primers for PCR were 5′- ACGTTGGATGTAGAAGTCCCCTTCCCTTAG -3′ and 5′- ACGTTGGATGGAACAAGGACTTTCGTCCTC-3′(reverse) for rs17321515, 5′- ACGTTGGATGACGAGCTTTGTGTCATGAGG- 3′, and 5′- ACGTTGGATGAGCTGCTGATGGTATTTTAC-3′ for rs2954029. PCR amplification was performed under the following conditions: an initial denaturation at 94 °C for 5 min, followed by 45 cycles of denaturing at 94 °C for 20 s, annealing at 56 °C for 30 s, and extending at 72 °C for 1 min. The genotypes of rs17321515 and rs2954029 were detected by direct DNA sequencing using the ABI veriti-384 Prism sequence detection system, and the raw data were analyzed using MassARRAY TYPER4.0 software (Agena, Inc). Genotyping was performed in blinded fashion and the success rates were > 95%.

### Statistical analysis

Statistical analysis was carried out using the statistical package for the social sciences (SPSS), version 22.0 (SPSS Inc. Chicago, IL, USA). The Hardy-Weinberg equilibrium between expected and observed genotype distribution and the distributions of genotype between patients and controls were analyzed by Pearson’s χ^2^ test. Baseline characteristics of each subjects were tested by t test and reported as mean ± standard deviation (S.D.). The Genotypes and allele frequencies were evaluated using the χ^2^ test, and the distributions between the NAFLD patients and the healthy controls were analyzed using the Fisher exact test or the Pearson χ^2^ test, where appropriate. The association between SNP and NAFLD risk was estimated by computing odds ratios (ORs) and 95% confidence intervals (CI) from the multivariate logistic regression analyses. *P* <  0.05 was considered as statistically significant.

## Results

### Clinical characteristics of the study population

The clinical characteristics of NAFLD patients and healthy controls are shown in the Table 1. The two groups were matched for gender and age (both *P* > 0.05). The NAFLD patients had higher BMI value and serum levels of ALT, GGT, TG, TC, LDL and FPG than healthy controls (all *P* <  0.05), besides, the serum level of HDL in NAFLD patients was significant low compared to the healthy controls (*P* <  0.05), No significant differences of serum AST, ALP, TBIL levels were observed between NAFLD patients and healthy controls (all *P* > 0.05).

**Table 1:** Clinical Characteristics of Patients with NAFLD and Healthy Controls^a^

	NAFLD patients (*n* = 146)	Controls (*n* = 175)	*P* Value
Female (%)	65 (44.52)	87 (49.71)	0.353
Age (y)	61.08 ± 15.70	60.15 ± 11.96	0.561
BMI (kg/m^2^)	26.93 ± 3.85	22.44 ± 2.88	< 0.001
ALT (U/L)	29.95 ± 17.69	22.21 ± 20.63	< 0.001
AST (U/L)	24.22 ± 9.01	22.88 ± 13.03	0.299
GGT (U/L)	39.56 ± 26.48	29.57 ± 36.35	0.006
ALP (U/L)	70.99 ± 16.69	73.75 ± 24.39	0.234
TG (mmol/L)	1.92 ± 1.31	1.33 ± 0.76	< 0.001
TC (mmol/L)	5.47 ± 0.82	4.24 ± 1.39	< 0.001
HDL (mmol/L)	1.20 ± 0.22	1.30 ± 0.37	0.001
LDL (mmol/L)	3.28 ± 0.53	3.11 ± 0.70	0.012
TBIL (umol/L)	13.15 ± 4.67	13.11 ± 5.18	0.942
FPG (mmol/L)	5.62 ± 1.93	4.63 ± 0.84	< 0.001

*Abbreviations: BMI* body mass index, *ALT* alanine aminotransferase, *AST* aspartate aminotransferase, *GGT* γ-glutamyltransferase, *ALP* alkaline phosphatase, *TG* triglyceride, *TC* total cholesterol, *HDL* high-density lipoprotein, *LDL* low-density lipoprotein, *TBIL* total bilirubin, *FPG* fasting plasma glucose, *NAFLD* nonalcoholic fatty liver disease

^a^Values are expressed as mean ± SD and compared by Student’s t-test

### Genotype and allele distribution of TRIB1 rs17321515 and rs2954029

The genotype distributions of TRIB1 rs17321515 and rs2954029 were in accordance with the Hardy-Weinberg equilibrium in both the NAFLD patients and healthy controls (all *P* > 0.05) (Table 2). As described in the Table 3, there were significant differences in the genotypes and alleles distribution of TRIB1 rs17321515 between NAFLD patients and controls group (*P* = 0.038; *P* = 0.026, respectively). The TRIB1 rs17321515 GA + AA genotype was the significant risk factor for the development of NAFLD (OR = 1.885; 95%CI: 1.157–3.070; *P* = 0.010), after adjusted for age, gender, and body mass index, the risk of TRIB1 rs17321515 GA + AA genotype was still marked (OR = 2.240; 95%CI: 1.196–4.197; *P* = 0.012) (Table 4). There was no significant difference in the genotype distribution of TRIB1 rs2954029, between NAFLD patients and controls group (*P* = 0.076), but a noteworthy difference of TRIB1 rs2954029 allele distribution was observed between NAFLD patients and controls group (*P* = 0.045) (Table 3). The TRIB1 rs2954029 TA + AA genotype was the significant risk factor for the development of NAFLD (OR = 1.627; 95%CI: 1.011–2.619; *P* = 0.045), after adjusted for age, gender, and body mass index, the risk of TRIB1 rs2954029 TA + AA genotype was still significant (OR = 2.050; 95%CI: 1.110–3.786; *P* = 0.022) (Table 4).

**Table 2:** Results of the Hardy-Weinberg Equilibrium^a^

Gene Locus	Groups	χ^2^	*P* Value
rs17321515	NAFLD patients	0.403	0.526
	Controls	1.693	0.193
rs2954029	NAFLD patients	0.041	0.840
	Controls	2.905	0.088

^a^Data were compared by chi-square test

**Table 3:** Distribution of Genotypes and Allele Frequencies of TRIB1 in NAFLD Patients and Controls^a,b^

	NAFLD patients	Controls	χ^2^	*P* Value
rs17321515				
Genotypes			6.599	0.038
GG	37 (26.6)	67 (40.6)		
GA	73 (52.5)	70 (42.4)		
AA	29 (20.9)	28 (17.0)		
Alleles			4.942	0.026
G	147 (52.9)	204 (61.8)		
A	131 (47.1)	126 (38.2)		
rs2954029				
Genotypes			5.160	0.076
TT	40 (27.4)	69 (39.4)		
TA	74 (50.7)	73 (41.7)		
AA	32 (21.9)	33 (18.9)		
Alleles			4.003	0.045
T	152 (52.4)	211 (60.3)		
A	138 (47.6)	139 (39.7)		

^a^Data were compared by chi-square test

^b^Values are expressed as No. (%)

**Table 4:** Association of Genotypes with NAFLD in the Study Group ^a^

	Unadjusted		Adjusted^a^	
	OR (95% CI)	*P* Value	OR (95% CI)	*P* Value
rs17321515				
GG	1		1	
GA + AA	1.885 (1.157–3.070)	0.010	2.240 (1.196–4.197)	0.012
rs2954029				
TT	1		1	
TA + AA	1.627 (1.011–2.619)	0.045	2.050 (1.110–3.786)	0.022

^a^Binary logistic regression model was adjusted for age, gender, and body mass index

### Association of the TRIB1 polymorphisms with clinical parameters in each group subjects

To investigate the potential correlation of TRIB1 polymorphisms with the clinical parameters in each study group, we compared the TRIB1 rs17321515 A allele and TRIB1 rs2953029 A allele with the clinical parameters of the overall series subjects, NAFLD patients and healthy controls. As shown in the Table 5, TRIB1 A allele carriers had the higher serum levels of GGT, TG, TC and LDL than non-carriers in the overall series (all *P* <  0.05). The higher serum level of LDL in the A allele carriers compared to the non-carriers in the NAFLD patients was observed (*P* <  0.05). In the healthy controls, the A allele carriers also had the higher serum TC level than non-carriers (*P* <  0.05). For the *TRIB1* rs2953029, there were higher serum levels of GGT and LDL in the A allele carriers than non-carriers in the overall series (both P <  0.05), and the serum level of TC in the A allele carriers was significant high compared to the non-carriers in the healthy controls (*P* <  0.05) (Table 6).

**Table 5:** Clinical Characteristics of *TRIB1* rs17321515 A Carriers and Non-Carriers in the Study Population^a^

Characteristic	Overall Series			NAFLD Patients			Controls		
	Carriers (*n* = 200)	Non-Carriers (*n* = 104)	*P* Value	Carriers (*n* = 102)	Non-Carriers (*n* = 37)	*P* Value	Carriers (*n* = 98)	Non-Carriers (*n* = 67)	*P* Value
Female, %	94 (47.0)	50 (48.1)	0.858	48 (47.1)	17 (45.9)	0.907	46 (46.9)	33 (49.3)	0.770
Age, y	60.40 ± 13.96	60.82 ± 13.73	0.801	60.37 ± 15.53	62.83 ± 16.14	0.399	60.42 ± 12.18	59.76 ± 12.29	0.735
BMI, kg/m^2^	24.68 ± 4.29	24.12 ± 3.57	0.255	27.01 ± 4.19	26.73 ± 2.86	0.702	22.27 ± 2.81	22.66 ± 3.02	0.397
ALT, U/L	27.27 ± 22.09	23.65 ± 14.97	0.093	30.23 ± 17.19	29.25 ± 19.07	0.766	24.18 ± 25.97	19.92 ± 9.90	0.143
AST, U/L	23.74 ± 11.88	23.19 ± 11.11	0.691	24.26 ± 8.57	24.12 ± 10.14	0.937	23.21 ± 14.57	22.50 ± 11.40	0.736
GGT, U/L	36.88 ± 37.78	29.70 ± 20.29	0.032	40.72 ± 26.74	36.68 ± 25.92	0.411	32.88 ± 46.39	25.70 ± 15.16	0.156
ALP, U/L	72.94 ± 20.38	70.80 ± 23.17	0.407	71.83 ± 15.31	68.90 ± 19.77	0.344	74.09 ± 24.61	72.08 ± 24.80	0.607
TG, mmol/L	1.98 ± 1.20	1.56 ± 1.07	0.003	1.95 ± 1.34	1.83 ± 1.24	0.599	2.33 ± 0.72	1.35 ± 0.86	0.893
TC, mmol/L	5.37 ± 1.40	4.58 ± 1.29	< 0.001	5.42 ± 0.80	5.60 ± 0.88	0.254	4.09 ± 1.21	3.93 ± 1.46	0.002
HDL, mmol/L	1.25 ± 0.29	1.25 ± 0.36	0.891	1.19 ± 0.21	1.21 ± 0.22	0.654	1.31 ± 0.34	1.29 ± 0.41	0.706
LDL, mmol/L	3.28 ± 0.68	2.91 ± 0.68	< 0.001	3.44 ± 0.59	3.32 ± 0.49	0.025	3.11 ± 0.77	3.09 ± 0.59	0.863
TBIL, umol/L	12.83 ± 4.62	13.56 ± 5.18	0.209	12.79 ± 4.36	14.06 ± 5.31	0.180	12.87 ± 4.90	13.16 ± 4.98	0.708
FPG, mmol/L	5.20 ± 1.70	4.91 ± 1.12	0.107	5.75 ± 2.14	5.30 ± 1.22	0.115	4.64 ± 0.71	4.65 ± 0.95	0.887

*Abbreviations: BMI* body mass index, *ALT* alanine aminotransferase, *AST* aspartate aminotransferase, *GGT* γ-glutamyltransferase, *ALP* alkaline phosphatase, *TG* triglyceride, *TC* total cholesterol, *HDL* high-density lipoprotein, *LDL* low-density lipoprotein, *TBIL* total bilirubin, *FPG* fasting plasma glucose, *NAFLD* nonalcoholic fatty liver disease

^a^Values are expressed as mean ± SD and compared by Student’s t-test

**Table 6:** Clinical Characteristics of TRIB1 rs2954029 A Carriers and Non-Carriers in the Study Population^a^

Characteristic	Overall Series			NAFLD Patients			Controls		
	Carriers (*n* = 212)	Non-Carriers (*n* = 109)	*P* Value	Carriers (*n* = 106)	Non-Carriers (*n* = 40)	*P* Value	Carriers (n = 106)	Non-Carriers (*n* = 69)	*P* Value
Female, %	97 (45.8)	55 (50.5)	0.424	44 (41.5)	21 (52.5)	0.233	53 (50)	34 (49.3)	0.925
Age, y	60.70 ± 13.79	60.08 ± 13.84	0.704	60.94 ± 15.60	61.39 ± 16.10	0.874	60.48 ± 11.90	59.64 ± 12.11	0.650
BMI, kg/m^2^	24.53 ± 4.29	24.26 ± 3.48	0.566	27.01 ± 4.22	26.74 ± 2.84	0.696	22.19 ± 2.79	22.83 ± 2.99	0.157
ALT, U/L	26.71 ± 21.78	23.97 ± 15.29	0.243	29.82 ± 17.00	30.23 ± 19.41	0.900	23.77 ± 25.21	19.82 ± 9.90	0.217
AST, U/L	23.68 ± 11.74	23.23 ± 10.92	0.738	24.12 ± 8.60	24.46 ± 10.02	0.836	23.27 ± 14.11	22.28 ± 11.24	0.623
GGT, U/L	36.53 ± 37.60	29.64 ± 20.11	0.035	41.06 ± 27.28	36.08 ± 24.46	0.304	32.26 ± 44.95	25.42 ± 15.25	0.151
ALP, U/L	73.09 ± 20.44	71.19 ± 23.03	0.452	71.59 ± 15.08	69.60 ± 20.09	0.516	74.51 ± 24.43	72.58 ± 24.46	0.611
TG, mmol/L	1.65 ± 1.17	1.49 ± 0.91	0.233	1.95 ± 1.35	1.83 ± 1.21	0.609	1.36 ± 0.87	1.28 ± 0.54	0.515
TC, mmol/L	4.71 ± 1.39	4.94 ± 1.78	0.142	5.44 ± 0.82	5.54 ± 0.84	0.535	4.57 ± 1.20	4.03 ± 1.47	0.008
HDL, mmol/L	1.26 ± 0.29	1.25 ± 0.35	0.721	1.20 ± 0.20	1.19 ± 0.24	0.923	1.32 ± 0.34	1.27 ± 0.40	0.376
LDL, mmol/L	3.17 ± 0.65	2.94 ± 0.72	0.003	3.23 ± 0.51	2.88 ± 0.54	0.056	3.12 ± 0.76	3.08 ± 0.59	0.705
TBIL, umol/L	12.90 ± 4.67	13.55 ± 5.47	0.268	12.72 ± 4.39	14.16 ± 5.18	0.092	13.06 ± 4.94	13.20 ± 5.56	0.860
FPG, mmol/L	5.16 ± 1.67	4.94 ± 1.18	0.225	5.74 ± 2.12	5.35 ± 1.37	0.272	4.61 ± 0.77	4.67 ± 0.94	0.656

*Abbreviations: BMI* body mass index, *ALT* alanine aminotransferase, *AST* aspartate aminotransferase, *GGT* γ-glutamyltransferase, *ALP* alkaline phosphatase, *TG* triglyceride, *TC* total cholesterol, *HDL* high-density lipoprotein, *LDL* low-density lipoprotein, *TBIL* total bilirubin, *FPG* fasting plasma glucose, *NAFLD* nonalcoholic fatty liver disease

^a^Values are expressed as mean ± SD and compared by Student’s t-test

## Discussion

NAFLD is one of the most prevalent chronic liver diseases in the world and causes related morbidity and mortality of HCC [25]. NAFLD is a complicated manifestation of metabolic syndrome which characterized with obesity, insulin resistant, type 2 diabetes mellitus, hyperlipidemia, the risk of NAFLD development is significant associated with the environmental factors and genetic factors and so on [26–28]. Single nucleotide polymorphism (SNP) as a significant genetic factor, plays an important role in the development of NAFLD [29]. Many variants in *PNPLA3*, *TM6SF2*, *MBOAT7*, *GCKR* and so on which are significant associated with the risk of NAFLD had been found by genome-wide association studies [30]. In this study, we investigated the relationship of *TRIB1* rs17321515 and rs2954029 gene polymorphisms with the risk of NAFLD in the Chinese Han population for the first time. We found that *TRIB1* rs17321515 A and rs2954029 A alleles were significantly associated with the risk of development of NAFLD, and the genotype distribution of *TRIB1* rs17321515 was significant different between NAFLD patients and healthy controls.

Tribbles-1 (TRIB1) is one of the member of tribbles family that was first identified in *Drosophila* and possesses the function to regulate the cell division and migration [12, 13], subsequent studies confirmed that *TRIB1* encodes a human homologue of the Drosophila tribbles protein and the association of *TRIB1* variant with the serum lipids metabolism was discovered by GWAS in the European population [14, 31]. In view of the new role of TRIB1 in lipoprotein metabolism, Burkhardt et al. investigated the role of hepatic TRIB1 on the serum lipoproteins in mice, they found the serum levels of TG and cholesterol were significant variational in the TRIB1-knockout mice or mice with TRIB1 hepatic-specific overexpression [32]. Accumulated evidences had been reported which focus on the significant role of *TRIB1* rs17321515 and rs2954029 in the development of NAFLD in different ethnic groups. In a Spanish familial hypercholesterolemia cohort study, the higher waist circumference and higher serum TG level were observed in the rs1732151 A/A carriers [33]. In a 10-year follow-up of the GLACIER study, Varga et al. reported that TRIB1 rs2954029 was strongly associated with the 10-year changes in lipid levels [34]. In consideration of deficient data of *TRIB1* rs17321515 and rs2954029 in the lipids metabolism in Chinese Han population, we investigated the relationship of *TRIB1* rs17321515 and rs2954029 with the risk of NAFLD and their effects on lipid profiles in Chinese Han population. We observed that *TRIB1* rs17321515 A and rs2954029 A alleles were significantly associated with the development risk of NAFLD, and the genotype distribution of rs17321515 was markedly different between NAFLD patients and healthy controls. In addition, rs17321515 A carriers possess the higher serum levels of GGT, TG, TC, and LDL in the overall series, higher LDL level in NAFLD patients and higher TC level in healthy controls. Similar, rs2954029 A carriers possess the higher serum levels of GGT and LDL in the overall series and higher TC levels in healthy controls, the results above-mentioned were consist with the previous studies which conducted in other countries.

There are some limitations of our study that must be acknowledged, 1) the lacking of liver biopsy is the main limitation of our study, however, liver biopsy is invasive, which may lead to a small risk of serious morbidity. Thereby, we used abdominal ultrasound to diagnose NAFLD in the present study; 2) all the included subjects in this study are Han nationality, our conclusion may not be applicable to other nationality absolutely; 3) the amount of subjects were still relative small, more subjects should be included in the future study.

## Conclusion

In summary, we investigated the relationship of *TRIB1* rs17321515 and rs2954029 with the development of NAFLD and lipids metabolism in Chinese Han population for the first time. Our results showed that *TRIB1* rs17321515 and rs2954029 were significant associated with the risk of NAFLD, and the rs17321515 A and rs2954029 A alleles affect the serum levels of multiple lipid profiles in included subjects. Further studies in larger and multiple ethnic populations are needed to confirm the present data.

## Abbreviations

AASLD: American association for the study of liver diseases
ALP: Alkaline phosphatase
ALT: Alanine aminotransferase
AST: Aspartate aminotransferase
BMI: Body mass index
CI: Confidence intervals
CVD: Cardiovascular disease
EDTA: Ethylene diamine tetraacetic acid
FPG: Fasting plasma glucose
GGT: Gamma-glutamyltranspeptidase
GWAS: Genome-wide association studies
HCC: Hepatocellular carcinoma
HDL: High density lipoprotein
LDL: Low density lipoprotein
NAFL: Non-alcoholic fatty liver
NAFLD: Non-alcoholic fatty liver disease
NASH: Non-alcoholic steatohepatitis
ORs: Odds ratios
PCR: Polymerase chain reaction
SNP: Single nucleotide polymorphism
TC: Total cholesterol
TG: Triglycerides
TRIB1: Tribbles-1
